# Supplementary material for: Weight Gain Predicts Metabolic Syndrome among North Korean Refugees in South Korea
Source: Int J Environ Res Public Health. 2021 Aug 11;18(16):8479. doi: 10.3390/ijerph18168479 (PMC8394171; doi:10.3390/ijerph18168479)
Supplement: Supplementary file 1 [file ijerph-18-08479-s001.zip › ijerph-1300102-supplementary.pdf]

Supplementary Table S1. Health questionnaire of NORNS study

**Questionnaire**	**Description**
Sociodemographic characteristics	Sex, age, education level in North Korea, main job in North and South Korea, marital status in North and South Korea, current income, resident area in North Korea, date of escape from North Korea, date of arrival in South Korea
Disease history	Disease diagnosed by medical doctor in South or North Korea
Health-related lifestyle	Smoking, alcohol drinking, exercise, nutritional supplements intake
Women health	Menstration status, pregnancy and delivery history
Mental health	Depression, psychological stress, suicidal trial, suicidal idea
